# Improving psychotropic drug prescription in nursing home patients with dementia: design of a cluster randomized controlled trial

**DOI:** 10.1186/1471-244X-13-280

**Published:** 2013-11-01

**Authors:** Claudia HW Smeets, Martin Smalbrugge, Debby L Gerritsen, Marjorie HJMG Nelissen-Vrancken, Roland B Wetzels, Klaas van der Spek, Sytse U Zuidema, Raymond TCM Koopmans

**Affiliations:** 1 Department of Primary and Community Care, Centre for Family Medicine, Geriatric Care and Public Health, Radboud university medical center, Code 117 ELG, P.O. Box 9101, 6500, HB Nijmegen, the Netherlands; 2 Department of General Practice and Elderly Care Medicine/EMGO + Institute for Health and Care Research, VU Medical Center, P.O Box 7057, 1007, MB Amsterdam, The Netherlands; 3 Dutch Institute for Rational Use of Medicine, P.O. Box 3089, 3502, GB Utrecht, the Netherlands; 4 Department of General Practice, University of Groningen, University Medical Center Groningen, HPC FA21, P.O. Box 196, 9700, AD Groningen, the Netherlands

**Keywords:** Dementia, Psychotropic drugs, Nursing homes, Medication safety, Clinical trial

## Abstract

**Background:**

Neuropsychiatric symptoms are highly prevalent in nursing home patients with dementia. Despite modest effectiveness and considerable side effects, psychotropic drugs are frequently prescribed for these neuropsychiatric symptoms. This raises questions whether psychotropic drugs are appropriately prescribed. The aim of the PROPER (PRescription Optimization of Psychotropic drugs in Elderly nuRsing home patients with dementia) II study is to investigate the efficacy of an intervention for improving the appropriateness of psychotropic drug prescription in nursing home patients with dementia.

**Methods/design:**

The PROPER II study is a multi-center cluster randomized controlled, pragmatic trial using parallel groups. It has a duration of eighteen months and four six-monthly assessments. Six nursing homes will participate in the intervention and six will continue care as usual. The nursing homes will be located throughout the Netherlands, each participating with two dementia special care units with an average of fifteen patients per unit, resulting in 360 patients. The intervention consists of a structured and repeated multidisciplinary medication review supported by education and continuous evaluation. It is conducted by pharmacists, physicians, and nurses and consists of three components: 1) preparation and education, 2) conduct, and 3) evaluation/guidance. The primary outcome is the proportion of patients with appropriate psychotropic drug use. Secondary outcomes are the overall frequency of psychotropic drug use, neuropsychiatric symptoms, quality of life, activities of daily living, psychotropic drug side effects and adverse events (including cognition, comorbidity, and mortality). Besides, a process analysis on the intervention will be carried out.

**Discussion:**

This study is expected to improve the appropriateness of psychotropic drug prescription for neuropsychiatric symptoms in nursing home patients with dementia by introducing a structured and repeated multidisciplinary medication review supported by education and continuous evaluation.

**Trial registration:**

Netherlands Trial Registry (NTR): NTR3569.

## Background

Neuropsychiatric symptoms (NPS) are highly prevalent in and burdensome for nursing home patients with dementia. Studies show prevalence rates of clinically relevant NPS of over 70% [1,2], and a cumulative two-year prevalence of even 97% [3]. NPS comprise a wide range of heterogeneous symptoms including delusions, hallucinations, agitation/aggression, depression, apathy, euphoria, anxiety, disinhibition, irritability, and aberrant motor behavior, which are frequently treated with psychotropic drugs. It is known that the efficacy of psychotropic drugs is limited and that their use is associated with considerable side effects such as extrapyramidal symptoms, somnolence, and increased risk for stroke, pneumonia, and mortality [4-7].

Nevertheless, the prevalence of psychotropic drug use (PDU) among nursing home patients with dementia is high with rates ranging from 48 to 66% [8-10]. Moreover, there is a risk for long-term use of psychotropic drugs whereas prescription for only a short period of time is recommended [4,11]. For instance, 74% of the nursing home patients with dementia use antipsychotics, anxiolytics, hypnotics, or sedatives for 83% of the duration of their stay [12], and 31% continue the use of antipsychotics, antidepressants, anxiolytics, hypnotics, anticonvulsants, or anti-dementia drugs throughout a 2-year period [9]. The contradiction of widely prescribed psychotropic drugs despite side effects and limited evidence for (long-term) effectiveness, suggests that psychotropic drugs may be prescribed inappropriately.

Systematic reviews on the effect of education, the involvement of pharmacists, and/or a multidisciplinary team show that these interventions may improve drug prescription in the elderly [13] or in nursing homes specifically [14,15]. For instance, improvements of about 30% in the prescription of drugs in nursing home residents [16,17], and discontinuation or dose reduction of antipsychotics in 61% of patients with dementia [18] have been found. Since the above-mentioned systematic reviews also include high quality studies not showing an effect, the authors suggest to focus in future studies on for example combining methods, multidisciplinary cooperation and direct communication between pharmacist, physician, and nurse, ways to improve the intervention, continuous education, and explicit procedures and routines for medication review. This encouraged us to develop an intervention integrating these elements into a new method of medication review. This medication review will be conducted face-to-face by a multidisciplinary team including not only the physician and pharmacist but also a member of nursing staff. Further, it will be supported by education on practical, organizational, and medical aspects, continuous evaluation, and will be repeated every six months. It is expected that the education and continuous evaluation offered to all participants gives each of them additional knowledge and structure for proper medication review with a specific emphasis on psychotropic drugs. Furthermore, the participation of nurses, through their daily observations representing the patient, and the face-to-face setting is expected to improve the quality of the review.

The PROPER II study (PRescription Optimization of Psychotropic drugs in Elderly nuRsing home patients with dementia) aims to study the effect of a structured and repeated multidisciplinary medication review supported by education and continuous evaluation on the appropriateness of PDU for treatment of NPS in nursing home patients with dementia. Secondary objectives are to investigate NPS, quality of life, activities of daily living, side effects and adverse events (including cognition, hospitalizations, and mortality).

## Methods/design

### Design and eligibility

The study is a multi-center, cluster randomized controlled, pragmatic trial using parallel groups, with a duration of eighteen months, and four six-monthly assessments. Six nursing homes will participate in the intervention and six will continue care as usual. Randomization will be conducted on the level of nursing homes to prevent contamination bias within the nursing home. The nursing homes will be located throughout the Netherlands, and each will participate with two dementia special care units (DSCUs). In the Netherlands, dementia patients usually reside on DSCUs, and medical care including prescription of psychotropic drugs is provided by an elderly care physician employed by the nursing home [19]. In an investigation preceding the PROPER II study, the observational PROPER I study, the same twelve nursing homes will participate. Nursing homes will be selected based upon their responses on a questionnaire regarding the proportion of patients using psychotropic drugs per individual DSCU. In order to maximize variation in the use of psychotropic drugs in the PROPER II study, those nursing homes, more specifically, those DSCUs with either high or low rates, will be approached for participation. Ideally, six nursing homes with high PDU, and six with low PDU will be included. Since the sample size needed for PROPER II (see below) is lower than for the PROPER I study [20], two DSCUs from each participating nursing home will be randomly included in the current study.

In total, 360 patients with a chart diagnosis of dementia will be included, i.e. on average fifteen patients of each of two DSCUs of twelve nursing homes. From DSCUs with more than fifteen patients, a random selection of fifteen patients will be included, regardless of their PDU. For DSCUs with less than fifteen patients, additional DSCUs will participate to retrieve the warranted number of patients per nursing home. Patients who die or are discharged from the DSCU, will be replaced during the study period. Physicians and nurses who are directly involved in the medical treatment and care for the patients will collect the patient data.

This study is a collaboration between the sections for elderly care medicine of three Dutch university Medical Centers and the Dutch Institute for Rational Use of Medicine [21], and is supported by the Dutch association for residential and home care organizations (ActiZ), and the Dutch Health Care Inspectorate.

### Intervention

**Figure 1:**
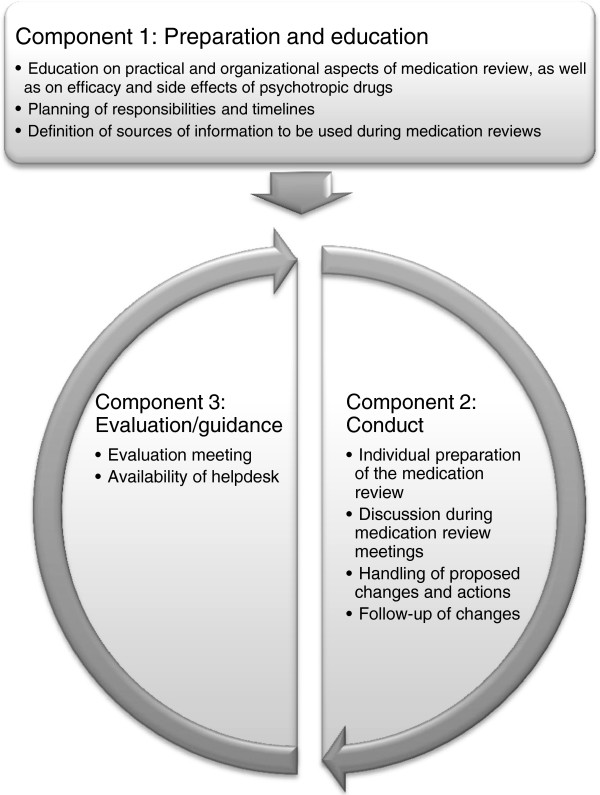
Intervention of the structured and repeated multidisciplinary medication review supported by education and continuous evaluation, consisting of three components.

The intervention consists of a structured and repeated multidisciplinary medication review supported by education and continuous evaluation. It consists of three components: 1) preparation and education, followed by a cycle of 2) conduct and 3) evaluation/guidance (Figure 1). A local project coordinator will be assigned to ensure appropriate planning and organization of these components. The first component takes place within one month after the baseline assessment of the trial; the second occurs within one month after the first component, or within one month after the evaluation/guidance meeting of the third component; the third component takes place within one month after the six- and twelve-month trial-assessments.

#### Component 1: preparation and education

The first component includes all preparations prior to the actual conduct of the medication review. The major part consists of an educational session. The education includes both the practical and organizational aspects of the medication review, as well as training about the efficacy and side effects of psychotropic drugs. It will be provided locally at each intervention nursing home and is to be attended by physicians, pharmacists, and nurses. The content is based upon the Guideline for problem behavior of the Dutch Association of Elderly Care Physicians and Social Geriatricians (Verenso) [22], and the Multidisciplinary guideline Polypharmacy in the elderly [23] including the STRIP method and Dutch versions of the START and STOPP tools [24]. The STRIP is the Systematic Tool to Reduce Inappropriate Prescribing and is a guidance for conducting structured medication reviews, the START is the Screening Tool to Alert doctors to Right Treatment, and the STOPP is the Screening Tool of Older Person’s potentially inappropriate Prescriptions. This education will be provided by the Dutch Institute for Rational Use of Medicine (IVM), which is specialized in the distribution of information and solutions for the proper, safe, affordable and effective use of medicine. The education is developed by the IVM in cooperation with the authors. Next to the education, this component comprises assigning responsibilities of the physicians, pharmacists, and nurses involved, timelines to be followed, and defining those sources of information that each of the participants will use for the medication review.

#### Component 2: conduct

The second component includes the actual conduct of the medication review and follow-up per individual patient. The structure is largely based on the STRIP [23]. The conduct of medication reviews per individual patient is a process of preparation, discussion on medication during the medication reviews, execution of the actions proposed, and evaluation of changes. The medication review will be conducted by a team consisting of an (elderly care) physician, pharmacist, and a nurse (assistant). Each of the participants will prepare the medication review. The physician is responsible for collecting medical data of the patient relevant for the discussion, such as type of dementia, comorbidity, and contraindications. The pharmacist is accountable for the actual medication list, knowledge on drug-drug interactions, and dosages. Whereas the STRIP involves the patient in the preparation of the medication review, the patient is in this study represented by the nurse. The nurse is therefore responsible for collecting information about the patient’s current behavior and potential PDU-related side effects and adverse events by means of completing a checklist per patient prior to the medication review. The medication review focuses on the appropriate prescription of psychotropic drugs for NPS, but also includes review of other drugs. During the discussion, the team determines whether (psychotropic) drugs must be additionally prescribed, tapered, discontinued, dose-adjusted, or replaced, and whether other actions are needed. These encompass additional diagnostics such as blood checks or electrocardiography, further observations of side effects and adverse events or NPS, referral to a psychologist or to a medical specialist, and use of psychosocial interventions by nursing staff in behavioral management. Proposed changes and actions will be registered and implemented after obtaining consent from the patient’s representative. (Non)compliance to the proposed actions is also registered. Further, changes in medication will be followed-up continuously by the physician and nurse.

#### Component 3: evaluation/guidance

Evaluation meetings regarding the conduct of the medication reviews will be organized to provide continous evaluation by guiding and counseling in the process of medication review. These meetings will be provided by the IVM and are to be attended by physician, pharmacist and nurse. Moreover, a help desk provided by the IVM is available for questions.

### Outcomes

#### Primary outcome

The primary outcome is the appropriateness of PDU defined as the proportion of patients with appropriate PDU. Assessment of appropriateness in this study is limited to antiepileptics, antipsychotics, anxiolytics, hypnotics/sedatives, antidepressants, and anti-dementia drugs prescribed for treatment of NPS in dementia, for sleep disturbances, and for delirium. Based on the Medication Appropriateness Index [25], a scale will be developed specifically for those psychotropic drugs used for treatment of NPS in nursing homes. Information will be included from the Guideline for problem behavior of the Dutch Association of Elderly Care Physicians and Social Geriatricians (Verenso) [22], the Guideline for diagnostics and medical treatment of dementia of the Dutch Geriatrics Society [26], the drug database of the Royal Dutch Pharmacists Association [27], and the ‘Farmacotherapeutisch Kompas’ [28], a reference of drugs available in the Netherlands published by the Dutch Health Care Insurance Board (CVZ).

#### Secondary outcomes

Secondary outcomes are the overall frequency of PDU, NPS, quality of life, activities of daily living, psychotropic drug side effects and adverse events (including cognition, hospitalizations, and mortality).

The overall frequency of PDU will be collected from the patients’ medical files or from (prints of) the electronic pharmacist information system and categorized using the Anatomical Therapeutic Chemical (ATC) classification [29] into the following therapeutic subgroups: antiepileptics (N03A), antipsychotics (N05A), anxiolytics (N05B), hypnotics and sedatives (N05C), antidepressants (N06A), and anti-dementia drugs (N06D).

NPS will be assessed using the Neuropsychiatric Inventory – Questionnaire (NPI-Q), the Cohen-Mansfield Agitation Inventory (CMAI), the Nijmegen Observer-Rated Depression scale (NORD), and the Minimum Data Set Depression Rating Scale (MDS-DRS). The NPI-Q [30] is a brief version of the Neuropsychiatric Inventory, which was developed for measuring NPS in dementia [31]. The NPI-Q consists of twelve items on NPS, each scored for occurrence (yes/no format), severity (three-point Likert scale), and associated caregiver distress of NPS (six-point Likert scale). A validated Dutch version will be used [32]. The CMAI is a questionnaire on 29 agitated behaviors reflecting physical aggression, physically nonaggressive behavior, and verbally agitated behavior. All items regard frequency of behavior using a seven-point Likert scale [33]. The (construct) validity of the Dutch version [34,35] and reliability [36] have been extensively studied. The NORD is a recently developed and promising Dutch questionnaire on occurrence (yes/no format) of five observable depressive symptoms, for screening of depression in nursing home residents with or without dementia [37]. The MDS-DRS is a seven-item observational instrument consisting of seven items on depression derived from the Minimum Data Set of the Resident Assessment Instrument (MDS-RAI) [38,39]. Each item is scored for frequency on a three-point scale. The Dutch version of this instrument was studied for validity and reliability and considered suitable for research in nursing homes [40].

Quality of life will be assessed using the Qualidem, a 37-item observational instrument consisting of nine subscales for measuring quality of life, each item is scored for frequency on a four-point scale. It was developed for Dutch nursing home patients with dementia and proven reliable and valid [41,42]. In order to allow proper interpretation of the Qualidem scores, the severity of dementia will be assessed using the Global Deterioration Scale, a staging instrument indicating cognitive deterioration in dementia [43]. Additionally, the Revised Index of Social Engagement (RISE) [44] will be assessed. This is an observational instrument with six dichotomous items on social behavior, which is considered to contribute to quality of life. The RISE is a revised version of the Index of Social Engagement [45], and is derived from the MDS-RAI [38,46].

Activities of daily living will be assessed using a questionnaire also derived from the MDS-RAI [47], of which validity and reliability of the Dutch version were established [40]. This scale has been adapted for the Dutch nursing home situation and scoring was simplified, resulting in a scale of twelve items to be scored on a four-point scale for level of independence, and a thirteenth item regarding change compared with the previous month (Joke Smallenburg, personal communication 2011).

Psychotropic drug side effects and adverse events will be assessed by symptoms and disorders related to PDU, cognition, hospitalizations, and mortality. A scale representing common symptoms and disorders related to PDU will be developed for this study, based upon the Udvalg for kliniske undersogelser side effect rating scale (UKU) [48]. Cognition will be assessed using the Severe Impairment Battery-8 [49], a brief version of the Severe Impairment Battery [50]. It was developed as a brief instrument for patients with severe Alzheimer’s disease and is sensitive to change over time. The SIB-8 was translated into Dutch for this study. Hospitalizations will be assessed by the number, indication, and duration as reported by the physicians, and mortality will be derived from the patients’ medical files.

All assessments will take place at baseline, six months, twelve months and eighteen months. An overview of the outcomes is shown in Table 1.

**Table 1:** Overview of outcomes, instruments, and assessor at baseline, six, twelve, and eighteen months

**Outcome**	**Instrument**	**Assessor**
Appropriateness of PDU	To be developed	Researcher
Frequency of PDU	Generic name and ATC code	Researcher
NPS		
NPS	NPI-Q	Nurse
Agitation/aggression	CMAI	Nurse
Depression	NORD	Nurse
Depression	MDS-DRS	Nurse
Quality of life		
Quality of life	Qualidem	Nurse
(For interpretation of Qualidem)	GDS	Physician
Social engagement	RISE	Nurse
Activities of daily living	Instrument derived from MDS-RAI	Nurse
Psychotropic drug side effects and adverse events		
Symptoms and disorders related to PDU	Instrument derived from UKU	Physician
Cognition	SIB-8	Physician/representative
Hospitalizations	Number, indication, and duration	Physician
Mortality	Occurrence	Researcher

*Abbreviations:PDU* psychotropic drug use, *ATC* Anatomical Therapeutic Chemical, *NPS* neuropsychiatric symptoms, *NPI-Q* Neuropsychiatric Inventory – Questionnaire, *CMAI* Cohen-Mansfield Agitation Inventory, *NORD* Nijmegen Observer-Rated Depression scale, *MDS-DRS* Minimum Data Set Depression Rating Scale, *GDS* Global Deterioration Scale, *RISE* Revised Index of Social Engagement, *MDS-RAI*: Minimum Data Set Resident Assessment Instrument, *UKU* Udvalg for kliniske undersogelser side effect rating scale, *SIB-8* Severe Impairment Battery-8.

#### Baseline characteristics

Other characteristics collected at baseline will be: age, sex, duration of nursing home admission, type of dementia as documented in the patients’ files, and comorbidity. Comorbidity will be assessed using a checklist on 25 chronic diseases considered most prevalent in a nursing home population. This checklist is a selection of those International Classification of Primary Care (ICPC) chronic diseases and comorbidities that are most prevalent in general practice [51], and adapted for the PROPER II study.

#### Process analysis

Also, a process analysis will be carried out on the actual use of the intervention and the factors determining its implementation, especially regarding facilitators and barriers. In addition, reasons for non-compliance with the intervention and time spent on medication review will be assessed, and the meetings guided by the IVM will be evaluated. Separate checklists for nurses, physicians, pharmacists, as well as for the nursing home’s local project coordinator will be used.

### Sample size

Assuming an increase in the proportion of patients with appropriate PDU from 60% to 80% in the intervention group and equal randomization to the intervention or control group, a significance level (alpha) of 0.05, a power of 80%, an average cluster size of fifteen patients per DSCU, and an ICC of 0.05 [52], a sample size of 21 clusters is sufficient to detect a statistically significant difference applying Russ Lenth software [53] and calculation methods according to Twisk [54]. Allowing for a DSCU drop-out of ten percent, in total 23 clusters are needed, resulting in the inclusion of two DSCUs in each of twelve nursing homes.

### Statistical analysis

Multilevel analysis will be applied to study the change in the proportion of patients with appropriate PDU between baseline and the average at six, twelve, and eighteen months on intervention DSCUs and control DSCUs, after correction of relevant covariates, such as baseline PDU and NPS. The use of a multilevel model will be applied for a number of reasons: patient PDU is hypothesized to be dependent on the prescription policy of the physician and thus to be nested within DSCUs, the longitudinal design and cluster randomization, and the replacement of drop-outs.

### Ethics approval

The local Medical Ethics Review Committee ‘CMO Regio Arnhem-Nijmegen’ rated the study (number 2012/226) and pronounced that the study is in accordance with the applicable rules in the Netherlands concerning the review of research ethics committees and informed consent. Representatives of all selected patients will be approached in writing to inform them about the study and to give them the explicit opportunity to refrain from participation of the patient in the study. The study will be conducted in accordance with the Declaration of Helsinki [55].

## Discussion

This protocol presents the design of a cluster randomized controlled trial evaluating the effectiveness of a structured and repeated multidisciplinary medication review supported by education and continuous evaluation to improve appropriate prescription of psychotropic drugs for NPS in nursing home patients with dementia.

The strength of this study’s intervention is the multidisciplinary three-component approach of involving professionals who are educated to carry out a structured and repeated medication review. By including not only the pharmacist and physician but also the nurse, the multidisciplinary team is expected to bring optimal knowledge from different perspectives. In this setting, not only medical and pharmaceutical expertise is taken into account, but also insight into the patients’ NPS, for which the psychotropic drugs are prescribed. Besides, the nurse has close contact with the representative of the patient, which further allows input on wishes regarding treatment or acceptation of NPS for the individual patient to be included in the medication review. Moreover, this study is a broad collaboration between several Dutch parties. Aside from the sections for elderly care medicine of three Dutch university Medical Centers, which have close connections with numerous nursing homes, the Dutch Institute for Rational Use of Medicine, the Dutch association for residential and home care organizations (ActiZ), and the Dutch Health Care Inspectorate are actively involved in this project. This has not only contributed to the design of the study and structure of the intervention, but will also facilitate the knowledge transfer of the results to daily practice after completion of the study. In case effectiveness of this three-component intervention is shown, this medication review method will be used on a broader scale to increase awareness of physicians, pharmacists and nurses of proper psychotropic drug use.

The study may have some limitations. Firstly, the involvement of a pharmacist for medication review is currently starting to become part of usual care, also in the control nursing homes. However, these medication reviews are most likely not introduced in a similar education-based, structured, and multidisciplinary fashion. Secondly, the turn-over of pharmacists, physicians, and/or nurses will affect the knowledge regarding the proposed conduct of the medication reviews, in case new staff did not attend the education sessions. However, due to the pragmatic design, the study will have a large external validity and it is expected that a potential effect is at least not overestimated.

Concluding, in the PROPER II study we target to improve the quality of pharmacological treatment of NPS of nursing home patients with dementia, by implementing a sound intervention of a structured and repeated multidisciplinary medication review supported by education and continuous evaluation.

## Competing interests

The authors declare that they have no competing interests.

## Authors’ contributions

SZ designed the study, DG and MS co-designed, and RK assisted in designing the study. The Dutch Institute for Rational Use of Medicine in cooperation with the authors designed the intervention. CS wrote the paper, and MS, DG, MN, RW, KS, SZ, and RK co-wrote the paper. All authors read and approved the manuscript.

## Pre-publication history

The pre-publication history for this paper can be accessed here:

http://www.biomedcentral.com/1471-244X/13/280/prepub
